# Metabolism of Low-Dose Inorganic Arsenic in a Central European Population: Influence of Sex and Genetic Polymorphisms

**DOI:** 10.1289/ehp.10026

**Published:** 2007-03-27

**Authors:** Anna-Lena Lindberg, Rajiv Kumar, Walter Goessler, Ranjit Thirumaran, Eugen Gurzau, Kvetoslava Koppova, Peter Rudnai, Giovanni Leonardi, Tony Fletcher, Marie Vahter

**Affiliations:** 1 Institute of Environmental Medicine, Karolinska Institute, Stockholm, Sweden; 2 DKFZ (German Cancer Research Centre), Heidelberg, Germany; 3 Institut für Chemie - Analytische Chemie, Karl-Franzens-Universität, Graz, Austria; 4 Environmental Health Centre, Cluj-Napoca, Romania; 5 State Health Institute, Banska Bystrica, Slovakia; 6 ‘Jozef Fodor’ National Centre of Public Health, Budapest, Hungary; 7 London School of Hygiene & Tropical Medicine, London, United Kingdom

**Keywords:** arsenic, *AS3MT*, blood, *GSTO1*, methylation, *MTHFR*, polymorphisms, sex, urine

## Abstract

**Background:**

There is a wide variation in susceptibility to health effects of arsenic, which, in part, may be due to differences in arsenic metabolism. Arsenic is metabolized by reduction and methylation reactions, catalyzed by reductases and methyltransferases.

**Objectives:**

Our goal in this study was to elucidate the influence of various demographic and genetic factors on the metabolism of arsenic.

**Methods:**

We studied 415 individuals from Hungary, Romania, and Slovakia by measuring arsenic metabolites in urine using liquid chromatography with hydride generation and inductively coupled plasma mass spectrometry (HPLC-HG-ICPMS). We performed genotyping of arsenic (+III) methyltransferase (*AS3MT*), glutathione *S*-transferase omega 1 (*GSTO1*), and methylene-tetrahydrofolate reductase (*MTHFR*).

**Results:**

The results show that the M287T (T→C) polymorphism in the *AS3MT* gene, the A222V (C→T) polymorphism in the *MTHFR* gene, body mass index, and sex are major factors that influence arsenic metabolism in this population, with a median of 8.0 μg/L arsenic in urine. Females < 60 years of age had, in general, higher methylation efficiency than males, indicating an influence of sex steroids. That might also explain the observed better methylation in overweight or obese women, compared with normal weight men. The influence of the M287T (T→C) polymorphism in the *AS3MT* gene on the methylation capacity was much more pronounced in men than in women.

**Conclusions:**

The factors investigated explained almost 20% of the variation seen in the metabolism of arsenic among men and only around 4% of the variation among women. The rest of the variation is probably explained by other methyltransferases backing up the methylation of arsenic.

Arsenic is a worldwide water contaminant, and chronic exposure has been associated with a large number of health effects, such as different forms of cancer, skin lesions, vascular diseases, liver- and neurotoxicity, and diabetes mellitus [Internal Agency for Research on Cancer 2004; World Health Organization (WHO) 2001]. A wide variation in susceptibility to various health effects has been reported (National Research Council 2001), which, in part, may be due to the marked variation in the metabolism of arsenic (Vahter 2002).

The classical pathway for the metabolism of inorganic arsenic (iAs) involves alternating reduction and oxidative methylation with only one end product, dimethylarsinate [DMA(V)] (Vahter 2002). Another—newly proposed—pathway suggests methylation of arsenic–glutathione complexes (Hayakawa et al. 2005) or arsenic bound to proteins (Naranmandura et al. 2006) with methyl-arsonate [MA(V)] and DMA(V) as end products. Both pathways involve methylation of arsenic via one-carbon metabolism with *S*-adenosyl methionine (SAM) as the methyl donor and requiring reduced glutathione (GSH) (Figure 1). GSH and probably other thiols serve as electron donors in the reduction reactions (Delnomdedieu et al. 1994a, 1994b; Scott et al. 1993), which are catalyzed by reductases.

Following exposure to iAs by ingestion or inhalation, DMA(V) is the major metabolite found in urine in most mammals, including humans, but MA(V) is found in human urine and very rarely in urine of other mammals (Vahter 2002). The metabolism of iAs involves both detoxification (methylation) and activation (reduction). Although MA(V) and DMA(V) have a lower toxicity than inorganic arsenic species (Hughes and Kenyon 1998), the trivalent methylated metabolites are more reactive and toxic than the other arsenic metabolites (Kligerman and Tennant 2006; Petrick et al. 2000; Schwerdtle et al. 2003a, 2003b; Styblo et al. 2000; Vega et al. 2001). A series of studies reported the presence of methylarsonite [MA(III)] and dimethylarsinite [DMA(III)] in urine (Aposhian et al. 2000; Le et al. 2000; Mandal et al. 2001; Valenzuela et al. 2005), although high concentrations are not likely to be found in urine because the high reactivity renders them to bind in tissues (Vahter 2002). In contrast, it is reasonable to assume that the total amount of MA in urine reflects the formation of the highly toxic MA(III) in the body. This might explain the observed increasing prevalence of arsenic-related toxic effects (e.g., skin lesions, skin cancer, bladder cancer, chromosome aberrations) with an increasing percentage of MA in urine (Chen et al. 2003a, 2003b, 2005; Del Razo et al. 1997; Hsueh et al. 1997; Maki-Paakkanen J 1998; Steinmaus et al. 2006; Yu et al. 2000). Furthermore, experimental animals, most of which methylate arsenic efficiently to DMA with essentially no MA excretion, show a faster overall excretion of arsenic than humans (Vahter 2002). Also, people with a small percentage of urinary arsenic as MA show less retention of arsenic than those with a higher percentage of urinary MA (Vahter 1999).

Thus, it is essential to determine the reasons for the marked variation in the metabolism of arsenic between individuals and population groups. One reason could be genetic polymorphisms in the regulation of enzymes involved in arsenic metabolism (Vahter 2000). One arsenic-reductase has been identified to be glutathione-*S*-transferase omega (*GSTO1*), which is able to reduce both MA(V) to MA(III) and arsenate [As(V)] to arsenite [As(III)] (Zakharyan et al. 2001). Recently, a SAM-dependent arsenic methyl-transferase isolated in rat was found to be a homolog of human arsenic (+III) methyl-transferase (*AS3MT*; previously called *Cyt19*) (Lin et al. 2002). Enzymes involved in the one-carbon metabolism could also indirectly influence the metabolism of arsenic, for example, methylenetetrahydrofolate reductase (*MTHFR*) (Figure 1).

Our aim in the present study was to elucidate the reasons for intraindividual variation in arsenic metabolism. Therefore, we studied the influence of age, sex, body mass index (BMI), genetic polymorphisms, and selenium status on the arsenic metabolite pattern in urine.

## Materials and Methods

### Study population

This study is part of a case–control study concerning cancer risks in relation to low-level arsenic exposure via drinking water in Central Europe: Arsenic Health Risk Assessment and Molecular Epidemiology (ASHRAM). Study areas were defined as certain counties in Hungary (Bacs, Békés, Csongrad, and Jazs-Nagykun-Szolnok), Romania (Bihor and Arad), and Slovakia (Banská Bystrica and Nitra) with known hotspots of arsenic in drinking water. The recruitment of skin, bladder, and kidney cancer cases and hospital-based controls with appendicitis, abdominal hernias, duodenal ulcer, cholelithiasis, and fractures, as well as the methods for collecting urine and blood samples, are described elswhere (Lindberg et al. 2006; Thirumaran et al. 2006). In short, all spot urine samples and whole blood samples, in spite of country, were collected and stored at −20°C and −80°C, respectively, until analysis. Informed consent was obtained from all participants, and the study was approved by the ethics committee of each hospital. In order to eliminate any potential bias caused by cancer, we selected controls for evaluation of factors that influence the metabolism of iAs.

There was a cluster of values close to 100% DMA at the lowest water arsenic concentrations. The intercept between the sum of urinary arsenic metabolites plotted against current water concentration was 2.5 μg/L, indicating a significant contribution of arsenic from food at the low exposure levels (Lindberg et al. 2006). Also, when performing speciation analysis in urine samples with very low concentrations, it is often only the major species that is above the limit of detection, leading to nearly 100% DMA in this case. Because of the influence of food and analytical precision on the percentage of DMA (%DMA) in the low concentration range, we decided to evaluate factors that influence the metabolite pattern only at concentrations > 2 μg/L, at which the influence from food was less obvious. Furthermore, 11 individuals were also excluded because they had high urinary arsenic concentrations and low water arsenic concentrations in combination with a high percentage of DMA in the urine, indicating a significant contribution to the exposure via food. The sample size after these exclusions was 415 individuals.

### Determination of arsenic compounds with HPLC-HG-ICPMS

The arsenic metabolites in urine were measured by an inductively coupled plasma mass spectrometer (ICPMS; HP 4500 or Agilent 7500cs; Agilent Technologies, Waldbronn, Germany) equipped with an integrated sample introducion system and a hydride generation (HG) accessory together with an Agilent 1100 chromatographic system equipped with solvent degasser, autosampler, and a thermostatted column. The method is described in more detail elswhere (Lindberg et al. 2006). We adjusted the arsenic concentrations in urine to the average specific gravity in the population (1.017 g/cm^3^) using a refractometer (Leica TS 400 Refractometer; Leica Microsystems Inc., Buffalo, NY, USA) to compensate for variation in dilution.

### Determination of selenium in blood

Selenium in whole blood was analyzed at a commercial laboratory (Analytica AB, Luleå, Sweden) with an inductively coupled plasma sector field mass spectrometer (ELEMENT, ThermoElectron; Finnigan MAT, Bremen, Germany) monitoring *m/z* 78 in high-resolution mode (delta m/m = 11,000). Samples were prepared by 25-fold dilution with ammonia:Triton X:EDTA:ethanol mixture (2%:0.0005%:0.0005%:4%) with addition of arsenic at 10 ng/mL for internal standardization. Calibration was performed externally with matrix-matched standards. Accuracy and precision of the method were controlled by analysis of a commercial reference material (Seronorm SN ok0336; SERO AS, Billingstad, Norway).

### Genotyping

DNA was isolated from blood samples from both cases and controls using Qiagen mini-preparation kits (Qiagen GmbH, Hilden, Germany) and genotyped for single nucleotide polymorphisms (SNPs) in the following genes: *MTHFR* (Unigene accession no. Hs.214142; National Center for Biotechnology Information 2007), *GSTO1* (Hs.190028), and *AS3MT* (Hs.34492). The SNPs selected for the analysis were non-synonymous with a minor allele frequency of at least 10%. We performed genotyping using the 5′ nuclease allelic discrimination assay (TaqMan) in 96-well format, as described previously (Thirumaran et al. 2006). TaqMan primers and probes were purchased from Applied Biosystems (Foster City, CA, USA) as “Assays-by-Design.” Primer and probe sequences used for genotyping are shown in Table 1. Polymerase chain reaction (PCR) was performed in a 5–10 μL volume reaction using 5 ng DNA as template, premade master mix, and 0.5× probe–primer mix. The initial temperature conditions for PCR were set at 50°C for 2 min and 95°C for 10 min, followed by 35–40 cycles at 92°C for 15 sec and 60°C for 1 min. Genotyping on amplified PCR products was scored by differences in fluorescent levels of VIC and FAM (both from Applied Biosystems) in plates read on an ABI PRISM 7900HT sequence detection system using SDS 1.2 software (Applied Biosystems). Postoperation data were transferred as Microsoft Excel data (Microsoft Corporation, Redmond, WA, USA) and converted into genotype information.

### Direct DNA sequencing

We randomly verified 4% of genotyping results from allelic discrimination assays by direct DNA sequencing. The sequencing reactions were performed using the BigDyeR Terminator Cycle sequencing kit (Applied Biosystems) in a 10-mL volume containing PCR product pretreated with ExoSapIT (Amersham Biosciences, Uppsala, Sweden) and a sequencing primer (Table 1). The temperature conditions set for sequencing reactions were 96°C for 2 min followed by 27 cycles at 96°C for 30 sec, 54°C for 10 sec, and 60°C for 4 min. Sequencing reaction products were precipitated with 2-propanol, washed with 75% ethanol, resuspended in 25 mL water, and loaded onto an ABI Prism 3100 Genetic Analyzer (Applied Biosystems). Primary sequencing data were analyzed using a sequence analysis program (Applied Biosystems).

### Statistical analyses

To evaluate whether selection in favor of a specific genotype had occurred, we assessed the Hardy-Wienberg equilibrium using allele frequencies. Statistica 7.1 for Windows (StatSoft Inc., Tulsa, OK, USA) was used to perform the statistical analyses. In the multivariate analyses using linear regression, the variables were natural log (ln)-transformed as needed to meet the requirement of equal variance and normal distribution of residuals. We used Spearman correlation (*r_s_*) when testing for univariate associations between continuous variables. Nonparametric tests (Mann-Whitney *U*-test and Kruskal-Wallis test) were used in testing for univariate differences between groups. We used one-way analysis of variance when testing for univariate differences between genotypes and Pearson chi-square when testing for differences between categorical variables. The multiple regression models, including all individuals, included variables that were significantly associated with any of the relative proportions of arsenic metabolites in the uni-variate tests. The multiple regression models stratified by sex included the variables that were significantly associated with any of the proportions of arsenic metabolites in the multiple regression models, including all individuals. Tests for collinearity were performed using tolerance. We included BMI in the models instead of body surface area (BSA) because BMI gave a higher coefficient of determination (*R*^2^) compared with BSA. When testing for differences in subgroups (except sex), we used analysis of covariance with dichotomized independent variables [age, above a mean of 60 years or < 60 years; BMI, > 25 kg/m^2^ and < 25 kg/m^2^, the limit of overweight defined by the WHO (2000)] adjusted for continuous covariates that were significant in the multiple regression analyses that included all individuals. We generally used *p* < 0.05 to indicate statistical significance, except *p* < 0.10 was used for some interactions.

## Results

### Descriptive

The characteristics of the participants are shown in Table 2. A total of 225 males and 190 females, 60 years of age on average, were included in the study. Twenty percent were current smokers (26% of the men and 14% of the women; *p* < 0.001). The current smokers were younger (mean age, 53 years) than ex-smokers and nonsmokers (mean age, 62 years and 65 years, respectively; *p* < 0.001). Of the participants, 83% had consumed any kind of alcohol (95% of the men and 70% of the women; *p* < 0.001). The average BMI and BSA were 27 kg/m^2^ and 1.9 m^2^, respectively. We found a positive correlation between selenium in whole blood and urinary arsenic (*p* < 0.001). The main reason for this correlation was that the individuals from Hungary had both higher urinary arsenic concentrations and higher blood selenium concentrations than individuals form Romania and Slovakia. Among the Hungarians, Romanians, or Slovakians, analyzed separately, we found no association between arsenic and selenium concentrations. The average proportions of iAs (%iAs), MA (%MA), and DMA (%DMA) in urine were 8.3%, 17%, and 73%, respectively (Table 2). However, there were wide variations. We were able to detect traces of MA(III) in only two samples (0.23 and 0.25 μg/L, respectively) corresponding to 0.89 and 6.6% of the total arsenic metabolites. The allele and genotype frequencies for the A222V (C→T) *MTHFR*, E429A (A→C) *MTHFR*, A140D (C→A) *GSTO1*, and M287T (T→C) *AS3MT* polymorphisms are shown in Table 2. The genotype distributions for all polymorphisms were in accordance with the Hardy-Weinberg distribution. Only three individuals were homozygous for the variant allele in the *AS3MT* gene and were therefore combined with the heterozygotes for further analyses. Genotype distributions were not associated with sex or country.

### Univariate analyses

Men had higher %iAs (*p* = 0.04) and %MA (*p* = 0.003), and lower %DMA (*p* = 0.008) than women. BMI was negatively associated with %MA (*p* = 0.001) and positively associated with %DMA (*p* = 0.003), but it was not associated with %iAs. Blood selenium was positively associated with %MA (*p* < 0.001) and negatively associated with %DMA (*p* = 0.007), but not with %iAs. Individuals homozygous for the variant allele for the A222V (C→T) *MTHFR* polymorphisms had lower %DMA (*p* = 0.002) and higher %MA than individuals homozygous for the wild-type allele (*p* = 0.01; Figure 2). Individuals heterozygous and homozygous for the variant allele in the M287T (T→C) *AS3MT* polymorphism had lower %DMA (*p* = 0.003) and higher %MA (*p* < 0.001; Figure 2). The concentration of total urinary arsenic, smoking, or alcohol use were not associated with %iAs, %MA, or %DMA.

### Multivariate analyses

We designed one model for each arsenic metabolite. Table 3 shows the results of the multiple regression analyses to test whether the distributions of urinary arsenic metabolites were dependent on sex, age, BMI, selenium, and gene polymorphisms in *MTHFR*, *GSTO1*, and *AS3MT*. Similar to the univariate assessment, %DMA was associated with polymorphisms in *AS3MT* and *MTHFR* genes, selenium, BMI, and sex in decreasing order. The %MA was associated with a gene polymorphism in *AS3MT,* selenium, BMI, sex, and a gene polymorphism in *MTHFR* in decreasing order. However, for %iAs the multivariate analyses showed only an association with sex and an almost significant association with a polymorphism in the *MTHFR* gene. For %MA, we found a significant interaction between BMI (two categories: > 25 kg/m^2^ and below < 25 kg/m^2^) and selenium (two categories: above the median of 99 μg/L and > 99 μg/L; *p* = 0.06). Also, the corresponding interaction for %DMA was near significance (*p* = 0.10).

To better understand the difference in methylation capacity between males and females, we repeated the multiple regression analyses stratified by sex (Table 4). The sex-specific models show that selenium, BMI, and *AS3MT* polymorphisms affect the distribution of urinary arsenic metabolites in males, but not in females. Also, in males, mutation in one allele in the *MTHFR* gene altered the pattern of urinary arsenic metabolites; however, for females, mutations of both alleles were required. Furthermore, to test whether the sex difference in methylation capacity was dependent on age or BMI, we used analysis of covariance. In those < 60 years of age, males had a higher %MA than females, but this was not the case for those > 60 years of age (Figure 3A). Furthermore, men of normal weight had a higher %MA than overweight or obese women (Figure 3B).

## Discussion

The results of the present study show that the M287T (T→C) polymorphism in the *AS3MT* gene, the A222V (C→T) polymorphism in the *MTHFR* gene, BMI, and sex are major factors that influence arsenic metabolism in this Central European population. This is the first study to our knowledge to elucidate factors influencing the metabolism of iAs at these low concentrations, where the influence of arsenic exposure on the methylation reactions is negligible. It is also the first to study arsenic-related polymorphisms in Europe.

We found the allele frequencies to be in accordance with those in other Caucasian populations, but different from those in several other populations (PharmGKB 2007a, 2007b, 2007c, 2007d). In contrast to our finding that carriers of the variant allele of the M287T (C→T) polymorphism of the *AS3MT* gene had higher %MA, previous studies in Mexico (Meza et al. 2005) and Argentina (Schläwicke Engström et al. 2006) have shown other SNPs in *AS3MT* gene to be associated with lower %MA. However, we did not find those SNPs in the present study. Thus, different SNPs in this gene influence the metabolism of iAs in different directions. It is unlikely that *AS3MT* is the only methyl-transferase that methylates arsenic, given that there are around 100 different methyltransferases identified in the human body (Martin and McMillan 2002). This is supported by a recent *in vitro* study in which the %DMA was reduced from 53% in the normal cell line to 11% in the cell line with silenced *AS3MT* expression (Drobna et al. 2006). Interestingly, we found that the M287T (C→T) polymorphism in *AS3MT* was not as important in women as it was in men, who also had higher %MA than women.

**Figure 1:**
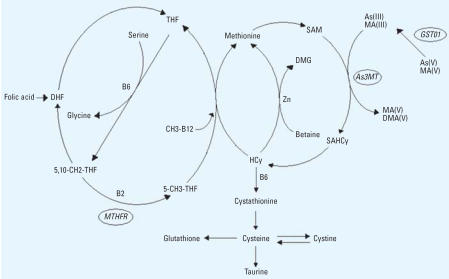
The methionine and folic acid cycles and the metabolic pathway of arsenic. Abbreviations: 5-CH3-THF, methyl tetrahydrofolate; 5,10-CH2-THF, methylenetetrahydrofolate; *As3MT*, arsenic (+3 oxidation state) methyltransferase; B2, vitamin B_2_ (riboflavin); B6, vitamin B_6_; CH3-B12, methylated vitamin B_12_; DHF, dihydrofolate; DMG, dimethylglycine; *GSTO1*, glutathione-*S*-transferase omega; HCy, homocysteine; SAHCy, *S*-adenosyl homocysteine; THF, tetrahydrofolate; Zn, zinc.

**Figure 2:**
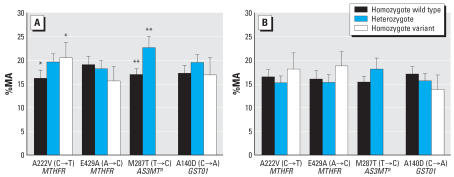
The mean and confidence interval of the %MA by genotype of *MTHFR* A222V, *MTHFR* E429A, *GSTO1* A140D, and *AS3MT* M287T for (*A*) males and (*B*) females. ^***a***^Heterozygotes and homozygotes for the varient allele in the *AS3MT* polymorphism are combined. *p = 0.01. **p < 0.001.

**Figure 3:**
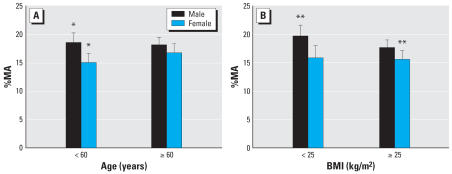
The mean and confidence interval of %MA by age (*A*) and BMI (*B*) in males and females. *p = 0.03.**p = 0.04.

Also, the A222V (C→T) polymorphism in the *MTHFR* gene was associated with higher %MA. *MTHFR* reduces methylenetetra-hydrofolate to methyltetrahydrofolate, which regenerates methionine from homocysteine in the one-carbon metabolism with methylated vitamin B_12_ as co-factor (Figure 1). The A222V (C→T) polymorphism has been associated with reduced enzyme activity and elevated levels of homocysteine (Baum et al. 2004), which in turn could lead to lower SAM-dependent methylation via feedback inhibition, possibly explaining our results. An interesting finding in the present study was that mutation on only one allele was needed to alter the arsenic metabolite pattern in males, whereas females needed mutations on both alleles. This discrepancy could be because females have a generally higher rate of remethylation of methionine from homocysteine than do males (Fukagawa et al. 2000). Furthermore, the influence of the A222V polymorphism on the metabolite pattern was more pronounced in individuals > 60 years of age (data not shown). The elderly are known to have poorer nutrition, especially lower vitamin B_12_ levels, than younger people (Martin 2006), which could make them more susceptible to the reduced enzyme activity that might result from the polymorphism.

Marnell et al. (2003) observed that two individuals with an uncommon genotype of *GSTO1* had an altered distribution of iAs metabolites in urine. However, studies with *GSTO1* knockout mice showed that they still reduced arsenic(V) species, but to a lesser extent (~ 20% of that found in wild-type mice) (Chowdhury et al. 2006). This animal study supports our finding that polymorphisms in *GSTO1* are not associated with an altered arsenic metabolite pattern, indicating nonenzymatic reduction and/or the presence of alternative enzymes for these reduction reactions.

Several studies have shown associations between malnourishment and increased risk for different arsenic induced health effects, partly due to less antioxidant defense and partly due to alterations in the metabolism of arsenic (Gamble et al. 2005; Milton et al. 2004; Mitra et al. 2004; Steinmaus et al. 2005). In the present study, we observed an increase in %DMA and a decrease in %MA and %iAs with increasing BMI. However, this association was probably not due to nutritional factors because most individuals were overweight (median BMI, 27 kg/m^2^). The BMI-related difference in methylation was mainly between normal-weight men and overweight and obese women, and we found a difference between the sexes in arsenic methylation only in individuals < 60 years of age; therefore, the results may suggest that sex steroids influence the methylation of arsenic. Estrogen is produced in adipose tissue in both males and females, leading to higher levels of estrogen in overweight individuals (Nelson and Bulun 2001). Furthermore, sex hormone-binding globulin is reduced with increasing body weight, leading to the release of free estrogen and progesterone (Pugeat et al. 1995). However, more studies are needed on differences between the sexes and the influence of sex steroids on the metabolism of arsenic.

Our hypothesis was that selenium would increase the methylation capacity, as shown in previous reports (Christian et al. 2006; Hsueh et al. 2003). However, the present study indicated the opposite association with selenium. When further evaluation of the interaction between selenium and BMI was performed, it appeared that Romanians and Slovakians had lower blood selenium but higher fractions of methylated metabolites of arsenic in urine compared with the Hungarians. Therefore, the association between %DMA or %MA and selenium was probably not a causal association, but rather an effect of, for example, different food habits in the different countries. The selenium concentrations (mean ± SD, 100 ± 22 μg/L) do not indicate deficiency, and the concentrations were in the same range as reported in several other European populations (Batáriová et al. 2005; Van Cauwenbergh et al. 1990).

We were not able to confirm previous findings that smoking and alcohol consumption negatively influence the metabolism of iAs (Hopenhayn-Rich et al. 1996; Hsueh et al. 2003). However, this was probably because of the low arsenic exposure in the present study.

Because previous reports have shown the presence of appreciable amounts of MA(III) in human urine and have claimed that improper sampling and storage conditions are likely reasons for the absence of MA(III) in urine (Feldmann et al. 1999), we also analyzed urine samples from patients treated with high levels of arsenic trioxide [30 mg arsenic/week (unpublished data)]. Spot urine samples were collected from four plasmacytoma patients both before and after treatment and immediately frozen in liquid nitrogen until analysis (~ 3 days after collection). A trace amount of MA(III), too low to be quantifiable, was found in one patient. The results of the present study indicate that MA(III) is not a significant metabolite in human urine, in contrast to previous reports (Aposhian et al. 2000; Le et al. 2000; Mandal et al. 2001; Valenzuela et al. 2005). Our interpretation is that the high reactivity renders them to bind in tissue (Vahter 2002).

In conclusion, the present study shows that polymorphisms in genes coding for enzymes involved in the metabolism of iAs explains a part of the large interindividual variation seen in the metabolism of iAs, especially in males, although not as much as we hypothesized. Other methyltransferases are probably backing up the methylation of arsenic. Genes coding for enzymes involved in the metabolism of iAs, as well as the influence of polymorphisms in these genes on metabolism, have only recently been investigated in epidemiologic studies. More large-scale studies are needed for complete understanding of arsenic metabolism.

## Figures and Tables

**Table 1:** Primers and probes used for SNP genotyping, including primer sequences, annealing temperatures, and fragment sizes of the amplified products used for PCR amplification and direct DNA sequencing.

Gene	Primers (5′→3′)	Probes (5′→3′)	Primer sequence	Temp (°C)	Size (bp)
*MTHFR* A222V (C→T)	F: GCACTTGAAGGAGAAGGTGTCT	VIC: ATGAAATCGGCTCCCGC	F: 5′-GAGGCTGACCTGAAGCACTTG-3′	60	200
	R: CCTCAAAGAAAAGCTGCGTGATG	FAM: ATGAAATCGACTCCCG	R: 5′-GTGGGGTGGAGGGAGCTTAT-3′		
*MTHFR* E429A (A→C)	F: GGAGGAGCTGCTGAAGATGTG	VIC: ACCAGTGAAGAAAGTGT	F: 5′-ATTCCTCTTCCCCTGCCTTTG-3′	59	198
	R: TGGTTCTCCCGAGAGGTAAAGA	FAM: CAGTGAAGCAAGTGT	R: 5′-TCCCCACTCCAGCATCACTC-3′		
*GSTO1* A140D (C→A)	F: GCCATCCTTGGTAGGAAGCTTTATT	VIC: AGAAGACTATGCTGGCCTA	F: 5′-GGGGGCCGATACAGTTAGC-3′	55	379
	R: TCGTTTACTCTGATGATAGCTAGGAGAAA	FAM: TAAAGAAGACTATGATGGCCTA	R: 5′-AGCAAGCCCATGACAAAGTCT-3′		
*AS3MT* M287T (T→C)	F: AATGGAGGAATTACAGGACATGAAAAAGA	VIC: ATTGGCATCAAACGTTAGT	F: 5′-GAGTGCTGGAGATGAACCGTGA-3′	56	231
	R: AGAAAGAATACCAGAAGTCATGGAAATTGT	FAM: TGGCATCAAACATTAGT	R: 5′-GGGCAAGAGCAGAAAGAATACCAGA-3′		

Abbreviations: F, forward; R, reverse; Temp, temperature,

**Table 2:** Participant characteristics, data on exposure, proportions of urinary arsenic species and genotype frequencies.

	No.	Percent	Median	10–90th percentile
Sex (male/female)	415	54/46		
Smoking (never/former/current)	415	48/31/20		
Alcohol use (yes/no)	412	83/17		
BMI (normal/overweight/obese)^a^	414	35/40/25	27	21–34
BSA^b^	414		1.9	1.6–2.1
Age (years)	415		61	44–75
Urinary arsenic (μg/L)^c^	415		8.0	2.7–38
%DMA	415		73	59–86
%MA	415		17	8.2–27
%iAs	415		8.3	2.4–19
Selenium in blood (μg/L)	377		99	74–126
*MTHFR* A222V (C→T)				
CC	181	44		
CT	190	46		
TT	43	10		
*MTHFR* E429A (A→C)				
AA	176	43		
AC	186	45		
CC	52	13		
*GST01* A140D (C→A)				
CC	182	44		
CA	190	46		
AA	42	10		
*As3MT* M287T (T→C)				
TT	324	79		
TC	84	20		
CC	3	1		

a Calculated as body weight (kg)/height (m)^2^; normal, 18.5–25 kg/m^2^; overweight, 25–30 kg/m^2^; obese, > 30 kg/m^2^.

b Calculated as body weight (kg)^0.425^ × height (cm)^0.725^ × 0.007184 (DuBois and DuBois 1916).

c Sum of iAs, MA, and DMA in urine.

**Table 3:** Multiple regression analyses to test whether %DMA, %MA, and %iAs are dependent on sex, age, BMI, selenium, and some polymorphisms.

		Sex^a^	Age (years)	BMI (kg/m^2^)	Selenium (μg/L)	*MTHFR* (CT vs. CC)	*MTHFR* (TT vs. CC)	*GSTO1* (CA vs. CC)	*GSTO1* (AA vs. CC)	*As3MT* (TC and CC vs. TT)
%DMA	B^b^	2.7*	0.021	0.36**	−0.082**	−0.57	−5.8**	−0.23	2.4	−4.7**
No. = 374; *R*^2^ = 0.10	Beta^c^	0.12*	0.021	0.15**	−0.15**	−0.025	−0.16**	−0.010	0.063	−0.17**
%MA	B	−2.2**	0.036	−0.27**	0.080**	1.3	3.4*	0.061	−1.9	4.7**
No. = 374; *R*^2^ = 0.15	Beta	−0.14**	0.051	−0.16**	0.21**	0.079	0.13*	0.0037	−0.071	0.23**
ln %iAs	B	−0.15*	−0.0053	−0.011	−0.000081	−0.017	0.20	−0.0075	0.012	0.061
No. = 347; *R*^2^ = 0.02	Beta	−0.11*	−0.094	−0.083	−0.0027	−0.013	0.10 (*p* = 0.08)	−0.0058	0.0057	0.038

a Male = 0; female = 1.

b Unstandardized regression coefficient.

c Standardized regression coefficient.

* *p* < 0.05.

** *p* < 0.01.

**Table 4:** Multiple regression analyses separated by sex to test whether %DMA, %MA, and %iAs are dependent on sex, BMI, selenium, and some polymorphisms.^a^

	No.	*R*^2^	Age^b^	BMI^c^	Selenium (μg/L)	*MTHFR* (CT vs. CC)	*MTHFR* (TT vs. CC)	*AS3MT* (TC and CC vs. TT)
%DMA								
Females	170	0.04	−0.015	0.072	−0.11	0.098	−0.20**	−0.065
Males	204	0.12	0.094	0.13*	−0.17**	−0.13*	−0.14*	−0.25**
%MA								
Females	170	0.04	0.12	−0.019	0.12	−0.049	0.14*	0.15*
Males	204	0.20	−0.039	−0.15**	0.27**	0.17**	0.11*	0.30**
ln %iAs								
Females	158	0.008	−0.038	−0.098	0.046	−0.049	0.17**	0.0070
Males	189	0.009	−0.14*	−0.10	−0.055	0.037	0.070	0.082

a Standardized regression coefficients (β).

b For age < 60 years, 0; for age ≥ 60 years, 1.

c For BMI < 25 kg/m^2^, 0; for BMI ≥ 25 kg/m^2^, 1.

* *p* < 0.10.

** *p* < 0.05.
